# Asthma Is an Independent Risk Factor for Acute Chest Syndrome in Children with Sickle Cell Disease in French Guiana

**DOI:** 10.3390/children11121541

**Published:** 2024-12-19

**Authors:** Gabriel Bafunyembaka, Mathieu Nacher, Chimène Maniassom, Archippe Muhandule Birindwa, Narcisse Elenga

**Affiliations:** 1 Department of Pediatrics, “Franck Joly” Hospital, Western French Guiana, Saint-Laurent du Maroni 97320, French Guiana; 2 Department of Pediatrics, Bukavu General Reference Hospital, GR4X+2VW Bukavu, Democratic Republic of the Congo; contact@univofbukavu.ac.cd; 3 Department of Paediatrics, Bukavu University Clinic, Bukavu Official University, GR2V+4Q4 N3 Bukavu, Democratic Republic of the Congo; 4 Clinical Investigation Center, Epidemiology/Public Health, Inserm 1424/CIC, Cayenne Hospital, Cayenne 97300, French Guiana; mathieu.nacher@ch-cayenne.fr; 5 Department of Pediatrics, Cayenne Hospital, Cayenne 97300, French Guiana; maniassomc@ch-cayenne.fr (C.M.); narcisse.elenga@ch-cayenne.fr (N.E.)

**Keywords:** sickle cell disease, asthma, acute chest syndrome, French Guiana

## Abstract

**Background/Objectives:** The overall incidence of asthma in children with sickle cell disease in French Guiana is unknown. Asthma is common in children with sickle cell disease and is associated with increased morbidity and mortality. This study aimed to describe the impact of asthma on the occurrence of acute chest syndrome in children with sickle cell disease who were followed up in French Guiana. **Methods:** We performed a multicenter nested case-control study between January 2012 and December 2022. Our study population consisted of children, aged between 6 months and 18 years, who were diagnosed with sickle cell disease at birth and hospitalized at least once for acute chest syndrome during the study period. **Results:** A total of 588 children were followed up for sickle cell disease. Of these, 390 had sickle cell disease, 180 had SC, and 18 had Sβ + thalassemia. Of the 390-sickle cell disease, we identified 35 who also had asthma, giving an estimated prevalence of asthma of 8.9% among children with sickle cell disease in French Guiana. Only asthma was significantly associated with acute chest syndrome (*p* < 0.001). **Conclusions:** The prevalence of asthma in children with sickle cell disease is underestimated. Asthma is an independent risk factor for acute chest syndrome. Given the seriousness of asthma in children with sickle cell disease, systematic screening for asthma in children with sickle cell disease has been implemented. This screening, which will be evaluated after one year, will help to better characterize asthmatic children with sickle cell disease and improve their care.

## 1. Introduction

Sickle cell disease (SCD) is the most common genetic disorder worldwide, affecting more than 50 million people, including 38 million in sub-Saharan Africa [1]. It is also the most frequently detected genetic disorder in the French population [2]. More than 300,000 children are born with SCD each year, and this number is expected to increase to 400,000 by 2050. The impact of this disease on global health is significant [3]. The pathophysiology of SCD is characterized by a vicious circle of four interrelated events: polymerization of hemoglobin S in a deoxygenated state, altered biorheology, increased vaso-occlusion by adhesion, hemolysis-mediated endothelial dysfunction, and concerted activation of sterile inflammation [4]. These molecular, cellular, and biophysical processes act synergistically to promote vaso-occlusive and hemolytic complications. These processes are responsible for acute and chronic pain as well as end-organ damage and failure. Acute hemolytic events include vaso-occlusive crisis (VOC) and acute chest syndrome (ACS). ACS is defined as an acute illness with a new segmental lung infiltrate consistent with consolidation but not atelectasis, together with one or more new respiratory symptoms or signs such as cough, chest pain, fever (>38.5 °C), hypoxemia (>3% decrease in oxygen saturation from a documented steady-state value on room air or oxygen saturation ≤ 94%), wheezing, and tachypnea [5]. More than half of children with homozygous SCD experience at least one episode of this complication during their first decade [6]. ACS is a life-threatening complication and the most common cause of death in SCD patients.

ACS is a common cause of acute lung disease in children with SCD. Asthma is also common in children with SCD and is associated with an increased incidence of ACS episodes and earlier death. The risk factors for asthma exacerbation and episodes of acute ACS are similar, and both can present with similar symptoms, such as coughing, wheezing, or chest pain. Despite the similarities between these two conditions, it is becoming increasingly clear that they are distinct entities. Asthma, as a comorbidity of SCD, is becoming better known, although there are still significant gaps. Asthma exacerbations in children with SCD have been associated with an increased incidence of ACS at a young age (median age 2.4 years) [6]. Several plausible explanations for the relationship between asthma and acute chest syndrome include ventilation-perfusion mismatch, synergistic inflammatory response, and vascular leakage [7,8]. The Department of Paediatrics early diagnosis of asthma in children with SCD may allow preventive measures to be taken to reduce the impact of the disease.

French Guiana is a French overseas territory in South America, where SCD is a major public health problem [9]. The population of African descent consists of three main groups, including Guianese Creoles, and Maroons (descendants of runaway slaves), and, more recently, Haitian immigrants [10]. The region is also characterized by health inequalities in terms of access to healthcare and inadequate health infrastructure compared to mainland France. The estimated incidence of SCD at birth is 1 in 227 [11] and the overall incidence of asthma in children with SCD is unknown. Under these conditions, asthma screening in children with sickle cell disease is challenging. Most importantly, an inventory of acute complications in patients with sickle cell disease can provide data on what already exists and can better guide preventive management. This study aimed to describe the impact of asthma on the occurrence of ACS in children with SCD who were followed up in French Guiana.

## 2. Materials and Methods

### 2.1. Materials

Using existing epidemiological data, we collated the medical records of children followed for sickle cell disease (SCD) at three public hospitals in French Guiana between January 2012 and December 2022. The study was based on data extracted from the Programme de Médicalisation des Systèmes d’Information (PMSI) and medical records of sickle cell children from various public hospitals in French Guiana (Centre Hospitalier de l’Ouest Guyanais Franck Joly, Centre Hospitalier de Kourou and Centre Hospitalier de Cayenne “Andrée Rosemon”).

### 2.2. Methods

This multicenter nested case-control study was conducted from January 2012 to December 2022. Our study population consisted of children from French Guiana aged between 6 months and 18 years, diagnosed with SCD at birth, and hospitalized at least once for acute ACS during the study period.

- Case inclusion criteria

SCD, aged between 6 months and 18 years, without asthma, was followed in one of the three public hospitals of French Guiana.

Diagnosed with asthma.

b. Control inclusion criteria

SCD, aged between 6 months and 18 years, was followed in one of the three public hospitals of French Guiana.

Had no asthma comorbidities.

Children without SCD were excluded from this study.

#### Selection Procedure

From the medical records of children with SCD collected during the study period, we were able to identify 35 children with SCD and comorbid asthma.

**Figure 1:**
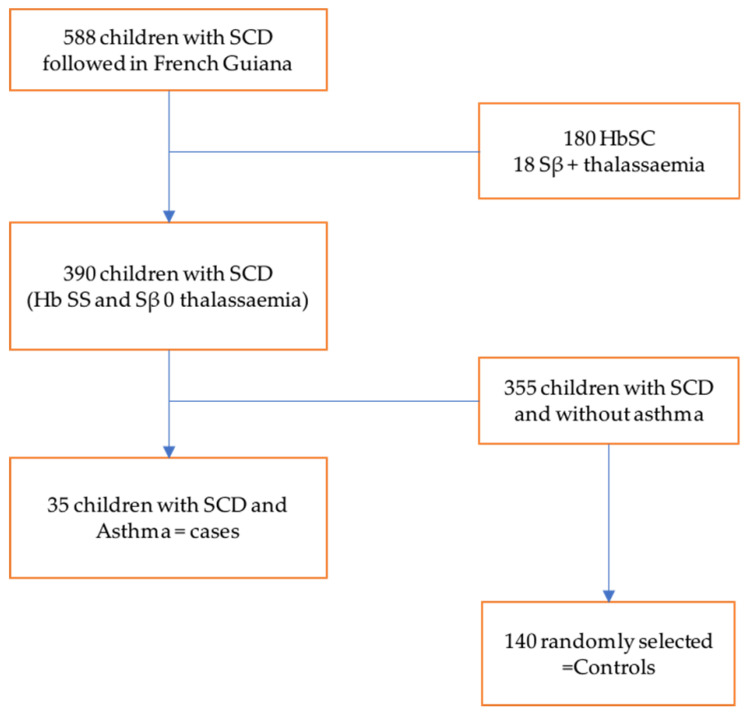
Flow chart of selected patients. There were 588 children with sickle cell disease monitored in French Guiana, including 180 children with SC profile and 18 with Sβ + thalassemia profile, and 390 children with SS and Sβ 0 thalassemia profile. Among the 390 children, we found 35 children with comorbid asthma. Of the remaining 355 sickle cell patients without asthma, 140 controls were selected after matching based on sex and age, i.e., 1 case for 4 controls.

To obtain our sample size, we randomly matched cases by age and sex, with one case for every four controls. The total sample size was 175, including 35 patients and 140 controls (see Figure 1).

The diagnosis of SCD was suspected at birth through universal newborn screening and confirmed by genetic testing at six months of age in all our patients, according to the medical records reviewed.

Asthma has been defined as follows:

- Any child under twelve months of age:

At least three episodes of wheezing dyspnea without specific atopic terrain, with good response to short-acting beta-2 mimetics, absence of pulmonary malformation on chest X-ray.

At least one to two episodes of wheezing dyspnea with a specific atopic background (prematurity with risk of bronchopulmonary dysplasia, eczema, allergic rhinitis, first-degree asthma), with good response to short-acting beta-2 mimetics, absence of pulmonary malformation on chest X-ray.

b. Any child aged twelve months or over:

Presenting with wheezing dyspnea, no pulmonary malformation on chest X-ray, good response to short-acting beta-2 mimetics.

Acute chest syndrome was defined (according to data documented in the medical record) as an episode of febrile respiratory distress with polypnoea, signs of respiratory distress, oxygen desaturation requiring oxygen spectacles or non-invasive ventilation, and new pulmonary infiltrate on the first chest X-ray or second chest X-ray comparing the image of the first and pulmonary infiltrate on a report.

### 2.3. Information Collected from Each Patient’s Medical Record

The following information was collected from medical records: family history of asthma or allergy, episodes of acute bronchiolitis, recent history of VOC, history of respiratory syncytial virus (RSV) infection, and cause and number of hospital admissions. Certain variables were retained as confounders: age, sex, place of residence, regularity of follow-up, history of VOC and RSV infections, and use of oral corticosteroids.

### 2.4. Statistical Methods

Categorical variables were summarized as numbers and proportions, while quantitative variables were summarized as means with standard deviation (if the distribution was symmetric) or as medians with minimum and maximum (if the distribution was asymmetric). The proportions of categorical variables were compared using the chi-square or Fischer test, depending on the conditions of use, and means and medians of quantitative variables were compared using Student’s *t*-test or the non-parametric Wilcoxon–Mann-Whitney test, respectively. A type I error threshold (alpha) of 5% was used for all the analyses. To identify factors associated with the occurrence of ACS, we constructed two simple and multiple linear regression models. As a measure of association, we present the unadjusted and adjusted beta coefficients with 95% confidence intervals. The threshold for statistical significance was set at 5%. To detect a difference in the frequency of hospitalisation for ATS between asthmatic and non-asthmatic SCD children with OR = 2, for a one-tailed test with 80% power, 5% alpha risk and an expected proportion of exposed controls of 20%, we performed a multiple matching procedure based on age and sex. All analyses were performed using the STATA 17th software (Stata Corp LP, College Station, TX, USA).

### 2.5. Ethical Approval Statement and Regulatory Aspects

The typology used in this study corresponds to research that does not involve human subjects (RnIPH). All data were collected from the medical records of the patients in the pediatric wards of the three hospitals. The participants were informed collectively by posters in the three hospitals, in the welcome booklet, and on the hospitals’ websites (general information on clinical research). The data were pseudonymized and processed by the pediatric staff of the Hospital Center of West Guiana (“Franck Joly”). The study was therefore an internal research study as defined by the French Data Protection Authority (Commission Nationale de l’Informatique et des Libertés–CNIL) and complied with the methodological requirements of the CNIL. Informed consent was not considered necessary as all patients were recruited retrospectively and anonymity of data collection was maintained.

## 3. Results

During the study period, 588 children were followed up for SCD. Of these children, 390 had SCD, 180 had SC, and 18 had Sβ + thalassemia. Of the 390 patients with SCD, we identified 35 who also had asthma, giving an estimated prevalence of asthma of 8.9% among children with SCD in French Guiana. The mean age of the study population was 8.41 ± 5.04 years. The majority of patients were from Saint-Laurent-du-Maroni in western French Guiana. Table 1 summarizes the sociodemographic characteristics of our study population, showing no statistically significant differences between cases and controls. Table 2 summarizes the risk factors associated with acute chest syndrome. Asthma was significantly associated only with ACS.

## 4. Discussion

The prevalence reported in our study may have been underestimated. Healthcare in French Guiana is limited [12]. Although care is free, there is only one pediatric pulmonologist, and many children with asthma remain undiagnosed. Asthma is a recognized comorbidity of SCD that affects 15–28% of children with SCD. We believe that young children with SCD have the same frequency of viral respiratory infections in early life as children of the same age in the general population do. These repeated viral infections in children with a family history of asthma provide a breeding ground for asthma treatment. Several studies have reported a higher prevalence than our own [13,14,15]. However, in all these studies, the definition of asthma was not clear. Often, it was a statement by the parents or doctor, or simply a review of the medical records revealed a history of asthma. In one prospective study, the diagnosis of asthma in children with SCD was based on three criteria: a parent with asthma, history of wheezing leading to breathlessness, and wheezing after exercise [13]. This model had a sensitivity of 100% when at least two of the criteria were present. If all studies used the same criteria to define asthma in patients with sickle cell patients, what would the prevalence have been? Taken together, these data highlight the clinical difficulty in diagnosing asthma and even asthma exacerbations in children with SCD. However, a confirmed diagnosis of asthma is associated with an increased incidence of acute ACS and VOC [16]. Therefore, it is important to diagnose asthma in all children with SCD to prevent its related complications.

Other studies have shown that asthma is an independent risk factor for ACS [17]. However, the mechanism by which asthma increases the risk of ACS is not known [16]. The lungs are among the main organs affected by SCD. It is the site of hypoxic and ischemic lesions, emboli due to bone marrow infarction, or fat necrosis, and has an increased propensity to develop pneumonia. Pulmonary function test abnormalities have been shown to be common in most patients with SCD, even in the steady state [18,19]. These abnormalities may be exacerbated in children with comorbid asthma, predisposing them to more complications such as ACS during severe VOC. The proximity of the two diagnoses sometimes raises the question of whether asthma and SCD are two different diseases or part of the same process [18]. Either way, children may present with viral syndrome or exacerbation of asthma that triggers regional hypoxia, sickle cell falcification, and ultimately systemic vaso-occlusion.

The differential diagnosis between an asthma attack and ACS in a child with SCD can be difficult. Indeed, there are situations in which asthma exacerbations may be misinterpreted as ACS episodes. Recognition of the clinical course and family history may allow the disease to be reinterpreted as asthma, thus modifying its clinical course. Many children with ACS do not benefit from rigorous asthma evaluation, including thorough personal and family history of asthma symptoms, pulmonary function testing, skin testing, and challenge testing [20]. Asthma is a comorbidity that needs to be taken seriously in children with SCD. It is not only associated with high comorbidity but also with increased mortality [20]. A few months ago, in French Guiana, we established a systematic screening system for asthma in children with SCD. This screening method is now being extended to everyone, including children living in remote communities.

To achieve this, training has been established for professionals working in remote centers. All children with a family history of asthma or signs of bronchial hyper-responsiveness were referred to the sickle cell reference center. This screening will be evaluated after one year. Other asthma screening programs have been published [21,22,23,24]. In any case, the impact of improved screening and treatment on pulmonary morbidity in SCD needs to be better defined, which is an area for future investigation.

## 5. Conclusions

The prevalence of asthma in children with SCD is underestimated. Asthma is an independent risk factor for ACS. Given the seriousness of asthma in children with SCD, systematic screening for asthma in children with SCD has been introduced. This screening, which will be evaluated after one year, will help to better characterize asthmatic children with sickle cell disease and improve their care.

## Figures and Tables

**Table 1:** Socio-demographic characteristics of cases and controls.

Variables	Total	Controls *n* = 140	Cases *n* = 35	*p* Value
Age (year)	8.41 ± 5.04	8.46 ± 5.09	8.17 ± 4.87	0.756
Gender (*n*, %)				
F	103 (49.05)	85 (48.57)	18 (51.43)	0.758
M	107 (50.95)	90 (51.43)	17 (48.57)	0.054
Place of residence (*n*, %)				
Kourou	24 (11.43)	16 (9.14)	8 (22.86)	
Cayenne	63 (30.00)	56 (32.00)	7 (20.00)	
Saint-Laurent-du-Maroni	123 (58.57)	103 (58.86)	20 (57.14)	

**Table 2:** Factors associated with Acute chest Syndrome.

Variables	OR naj (IC 95%)	*p* Value	OR aj (IC 95%)	*p* Value
Age (year)	0.95 (0.90; 1.01)	0.127	0.94 (0.87; 1.00)	0.079
Gender (*n*,%)				
F	1		1	
M	1.26 (0.71; 2.23)	0.417	0.63 (0.32; 1.25)	0.191
Frequency of ACS				
Controls	1		1	
Cases	18.41 (6.72; 50.43)	<0.001	18.47 (6.37; 53.56)	<0.001
Place of residence				
Kourou	1		1	
Cayenne	0.47 (0.17; 1.24)	0.131	0.66 (0.19; 2.24)	0.514
Saint-Laurent-du-Maroni	0.65 (0.27; 1.59)	0.354	0.94 (0.31; 2.84)	0.914
Sickle cell disease follow-up quality				
Regular	1		1	
Irregular	1.56 (0.86; 2.80)	0.137	0.80 (0.38; 1.70)	0.574
Previous history				
VOC	1		1	
RSV Infection	0.93 (0.52; 1.68)	0.824	0.89 (0.45; 1.75)	0.748
Corticosteroid	-		-	

OR naj—unadjusted linear regression coefficient; OR aj—adjusted linear regression coefficient; RSV—respiratory syncytial virus; ACS—acute chest syndrome; VOC—vasoocclusive crisis.

## Data Availability

The data are available from the corresponding author upon request.
